# Engagement with community liver disease management across the UK: a cross-sectional survey

**DOI:** 10.3399/BJGPO.2021.0085

**Published:** 2021-08-04

**Authors:** Helen Jarvis, Jonathan Worsfold, Vanessa Hebditch, Stephen Ryder

**Affiliations:** 1 Faculty of Medical Sciences, Newcastle University, Newcastle upon Tyne, UK; 2 British Liver Trust, Bournemouth, UK; 3 NIHR Nottingham Biomedical Research Centre at Nottingham University Hospitals NHS Trust, Nottingham, UK

**Keywords:** primary health care, liver diseases, quality of health care, access and evaluation, surveys and questionnaires

## Abstract

**Background:**

Liver disease is an increasing cause of premature mortality in the UK. Its management in primary care is not well understood. It is unclear what role commissioning bodies are playing in liver disease in the UK.

**Aim:**

To assess the level of engagement with community chronic liver disease management among clinical commissioning groups (CCGs) and health authorities across the UK.

**Design & setting:**

A cross-sectional survey to all UK CCGs and health authorities.

**Method:**

Survey questions were developed by the British Liver Trust, in collaboration with topic experts, and evaluated structures in place relating to liver disease management at commissioning and health board level.

**Results:**

There were 159 responses representing 99% UK coverage of CCGs and health boards. Twenty per cent reported an individual responsible for liver disease within their organisation, with 40% and 29% reporting having pathways in place to respond to abnormal liver blood tests and liver disease more generally, respectively. All those reporting use of pathways reported using national guidelines to guide content. Twenty-five per cent made use of transient elastography (FibroScan) and 16% of direct serum fibrosis markers (for example, enhanced liver fibrosis [ELF] score), which are both part of current National Institute for Health and Care Excellence (NICE) guidelines. There was marked regional variation in all areas of engagement surveyed, with Wales having exceptionally high levels of engagement in all areas in contrast to the other nations.

**Conclusion:**

The results of this survey should be used as a catalyst to highlight necessary regional improvements to the primary care management of chronic liver disease across the UK.

## How this fits in

Liver disease morbidity and mortality is increasing in the UK. GPs report a gap in knowledge and confidence in managing liver disease and it is unknown how commissioners are engaging with liver disease management. This study reports that commissioner or health board engagement with chronic liver disease shows unacceptable variation across the UK. The results should be used by clinicians and policymakers to improve primary care management of liver disease and reduce inequalities in care.

## Introduction

Liver disease is a common and increasing cause of morbidity and premature mortality in the UK and globally.^1^ This is in contrast to other common chronic diseases such as heart disease where reduction in morbidity and mortality has been seen over the past 50 years.^2^ The main causes of liver disease in the UK are alcohol-related liver disease (ARLD) and non-alcohol-related fatty liver disease (NAFLD), which is increasing in parallel with the obesity and type two diabetes mellitus (T2DM) epidemics seen in the UK and across the globe.^3^ As such, the risk factors for liver disease are in common with those for many other chronic diseases and cancers.

Chronic disease management is one of the cornerstones of primary care work, with management pathways, as well as diagnostic and treatment services, available locally and often incentivised nationally in the UK.^4^ This has led to more standardised evidence-based care for people living with T2DM, heart disease, and a range of other chronic conditions. At a practice level, this has led to widespread use of protocols and templates, with primary care nursing teams often being able to competently take the lead in chronic disease management along with high confidence and knowledge in these areas of clinical practice.^5,6^

Health care is organised differently in the four nations of the UK. England has CCGs, with the devolved nations maintaining the health board or authority model. These organisations fulfil a similar regulatory and oversight function for their population and support this effective community management of chronic disease, with disease or system-specific clinical leads to drive decisionmaking around diagnostics, referral pathways, and community interventions to standardise evidence-based care.

In sharp contrast to other disease areas, many GPs report a gap in confidence and knowledge when it comes to managing chronic liver disease.^7^ It is unclear if national guidelines,^8,9^ diagnostic tests, and exemplar pathways^10,11^ have become normalised and part of general practice in liver disease management across the UK. There is a notable lack of incentivisation for managing liver disease as a chronic disease across most of the UK, with no current or historical Quality and Outcome Framework (QOF) targets for liver disease.^12^

The aim of this study was, therefore, to assess the levels of engagement with chronic liver disease management among primary care commissioning bodies and health authorities across the UK. The primary objectives were first to ascertain whether structures and named decisionmakers were in place specific to liver disease management, and second whether guidelines were being promoted, including the evidence-based use of nationally recommended diagnostic tools.

## Method

An online cross-sectional survey was sent out to all UK commissioning bodies and health authorities between June and October 2020. All English CCGs and devolved nation equivalent health boards were included as participants in the study if a named contact could be identified from publicly available listings.

Questions in the survey evaluated structures and processes in place relating to liver disease detection and management at commissioning or health board level. The full survey content is available in Supplementary file 2. The survey was sense-checked on a small number of recipients before wider rollout. Owing to the initial low response rate, further responses were obtained using a freedom of information request (or equivalents in the devolved nations). At this stage responders were given the choice to respond to the online survey or fill in their responses on a Word document version of the survey.

Responses were collated from both collection methods and entered onto a Microsoft Excel spreadsheet by JW (checked by HJ) for analysis. A sample of the responses (*n* = 15/159, 9.4%) was cross-checked by direct contact with liver specialists in hospitals working within areas covered by some of the CCGs or health boards to confirm accuracy of any commissioned service reported from a provider perspective. Data analysis was carried out by HJ (checked by JW) using Microsoft Excel statistical software (2016).

The survey content development was led by the British Liver Trust, the largest charity representing people living with liver disease in the UK, in collaboration with hepatology and primary care experts. As such, this survey was developed and operationalised with patient and public involvement throughout, with equality of input from professionals and public representatives.

## Results

There were 159 responses to the survey, representing a 99% UK coverage of all UK CCGs and health boards. There was no response from two Scottish health boards. The survey covered three main areas of engagement. The first addressed structural workforce and processes in place specific to liver disease; the second focused on the use of recommended guidelines and diagnostic tools to detect liver disease; and the third concerned engagement with more proactive risk factor-based detection of liver disease.

### Workforce and processes in place specific to liver disease

**Figure 1.**
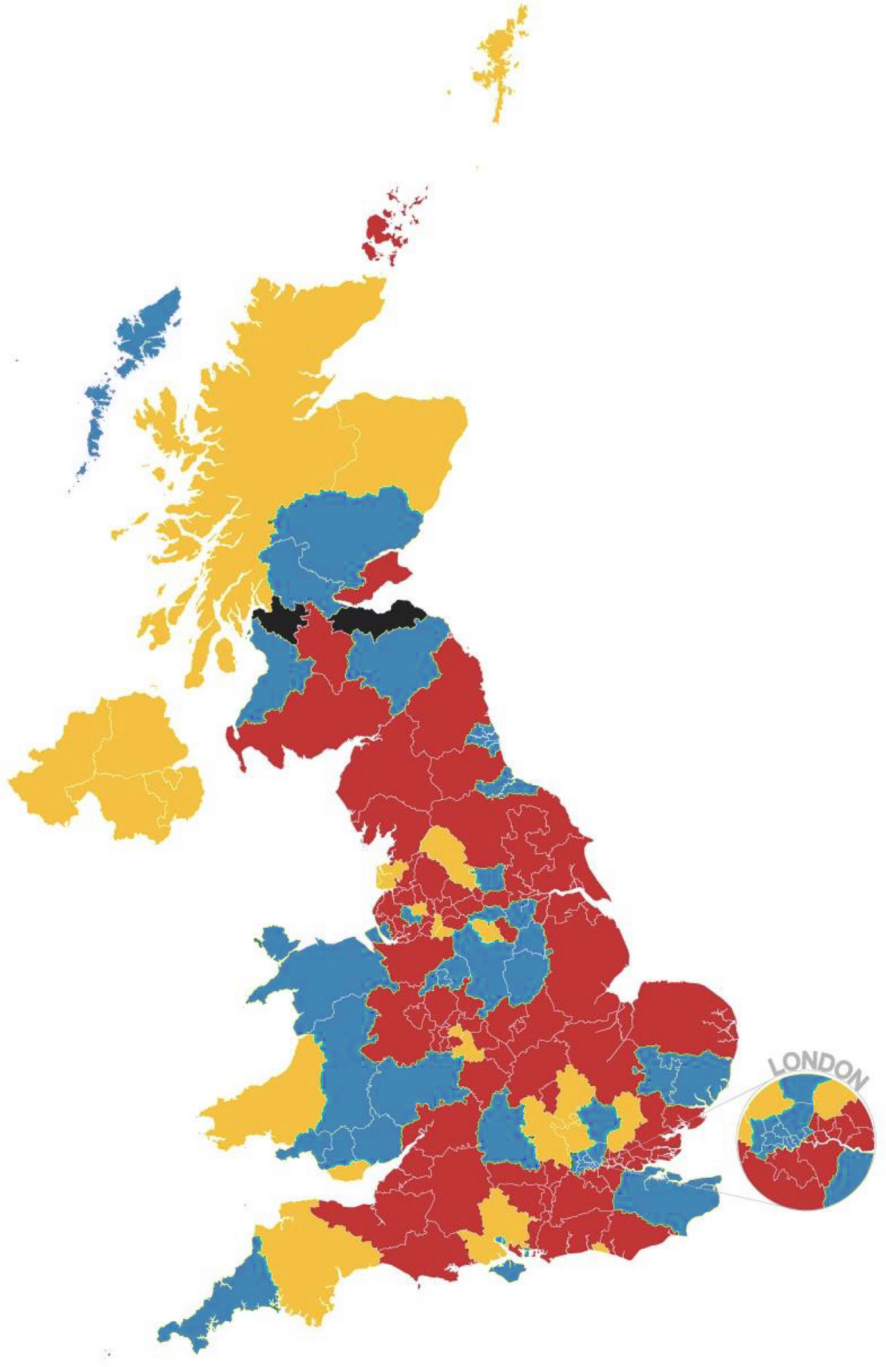
The availability of clinical commissioning group or health board-endorsed community liver pathways in the UK. Key: red = no pathway for either the interpretation of liver blood tests or liver disease more generally; yellow = pathway for the interpretation of liver blood tests only or pathways in development; blue = pathways for both; black = no response to survey.

UK wide, only 20% of CCGs and health boards questioned reported having a named individual within their organisation responsible for liver disease. Only 40% had an endorsed pathway in place for acting on liver blood test results, with even fewer having pathways in place for other aspects of liver disease management. Figure 1 provides a geographical overview of the provision of community liver pathways in the UK.

Very few survey responders (14%) were aware of any processes in place to monitor the adoption and efficacy of endorsed pathways. Just over one-third reported monitoring current statistics relating to liver disease locally. Further breakdown of these responses is shown in Table 1. There was a marked variation across the nations in the UK, with Wales standing out as having prioritised workforce and processes focusing on liver disease within their health boards. Eighty-six per cent of health boards in Wales had a named liver lead in place, in stark comparison with the 20% UK average; an indication of the increased priority given to this disease area.

**Table 1.** UK clinical commissioning group or health authority structures in place relating to liver disease

	Named person responsible for liver disease*n* (%)	Pathway for assessing abnormal LFTs*n* (%)	Pathway for liverdisease more generally*n* (%)	Processes in place tomonitor adoption and efficacy of pathway*n* (%)	Monitoring of current local statistics relating to liver disease*n* (%)
England*n* = 135	20 (15%)	49 (36%)	36 (27%)	21 (16%)	45 (33%)
Northern Ireland*n* = 5	0 (0%)	0 (0%)	0 (0%)	0 (0%)	0 (0%)
Scotland*n* = 12	6 (50%)	8 (67%)	5 (42%)	0 (0%)	6 (50%)
Wales*n* = 7	6 (86%)	7 (100%)	5 (71%)	1 (14%)	4 (57%)
UK total*n* = 159	32 (20%)	64 (40%)	46 (29%)	22 (14%)	55 (35%)

LFTs = liver function tests. *n* = number of commissioning bodies.

### The use of recommended guidelines and diagnostic tools to detect liver disease

Of those with pathways in place for liver disease, the vast majority were utilising pathways in line with current British Society of Gastroenterology (BSG) national guidelines with similar percentages of around 40% reported for both questions. Use of the indirect serum fibrosis markers, most commonly Fib-4 score and the NAFLD fibrosis score, were reported as being the most common CCG and health board-endorsed method of assessing for liver fibrosis. There was, however, marked regional variation with half (50%) of Scottish health boards utilising NICE-recommended direct serum markers (ELF test in the vast majority) in contrast with the 16% UK average, and 100% of Welsh health boards using transient elastography (FibroScan) as part of their endorsed pathways in contrast with one-quarter (25%) across the UK. The variable use of these diagnostic methods in detailed in Table 2.

**Table 2.** The recommended management of liver disease in UK clinical commissioning groups and health authorities in relation to national standards and guidelines

Area of UK	Using BSG-endorsed LFT pathway*n* (%)	Using liver fibrosisassessment*n* (%)	Using indirect serum fibrosis markers^a^ *n* (%)	Using direct serum fibrosis markers^b^ *n* (%)	Using transientelastography^c^ *n* (%)
England*n* = 135	50 (37%)	52 (39%)	56 (41%)	18 (13%)	26 (19%)
Northern Ireland*n* = 5	0 (0%)	0 (0%)	0 (0%)	0 (0%)	0 (0%)
Scotland*n* = 12	8 (67%)	8 (67%)	9 (75%)	6 (50%)	7 (58%)
Wales*n* = 7	6 (86%)	7 (100%)	5 (71%)	2 (29%)	7 (100%)
UK total*n* = 159	64 (40%)	67 (42%)	70 (44%)	26 (16%)	40 (25%)

^a^Included use of Fib4 score, non-alcoholic fatty liver diease (NAFLD) fibrosis score and AST:ALT (aspartate aminotransferase: alanine aminotransferase) ratio (other options given); ^b^All but one response was for the enhanced liver fibrosis (ELF) test, single response hyaluronic acid.^c^All using FibroScan.

BSG = British Society of Gastroenterology. LFT = liver function test. *n* = number of commissioning bodies

### Engagement with more proactive risk factor-based detection of liver disease

**Figure 2.**
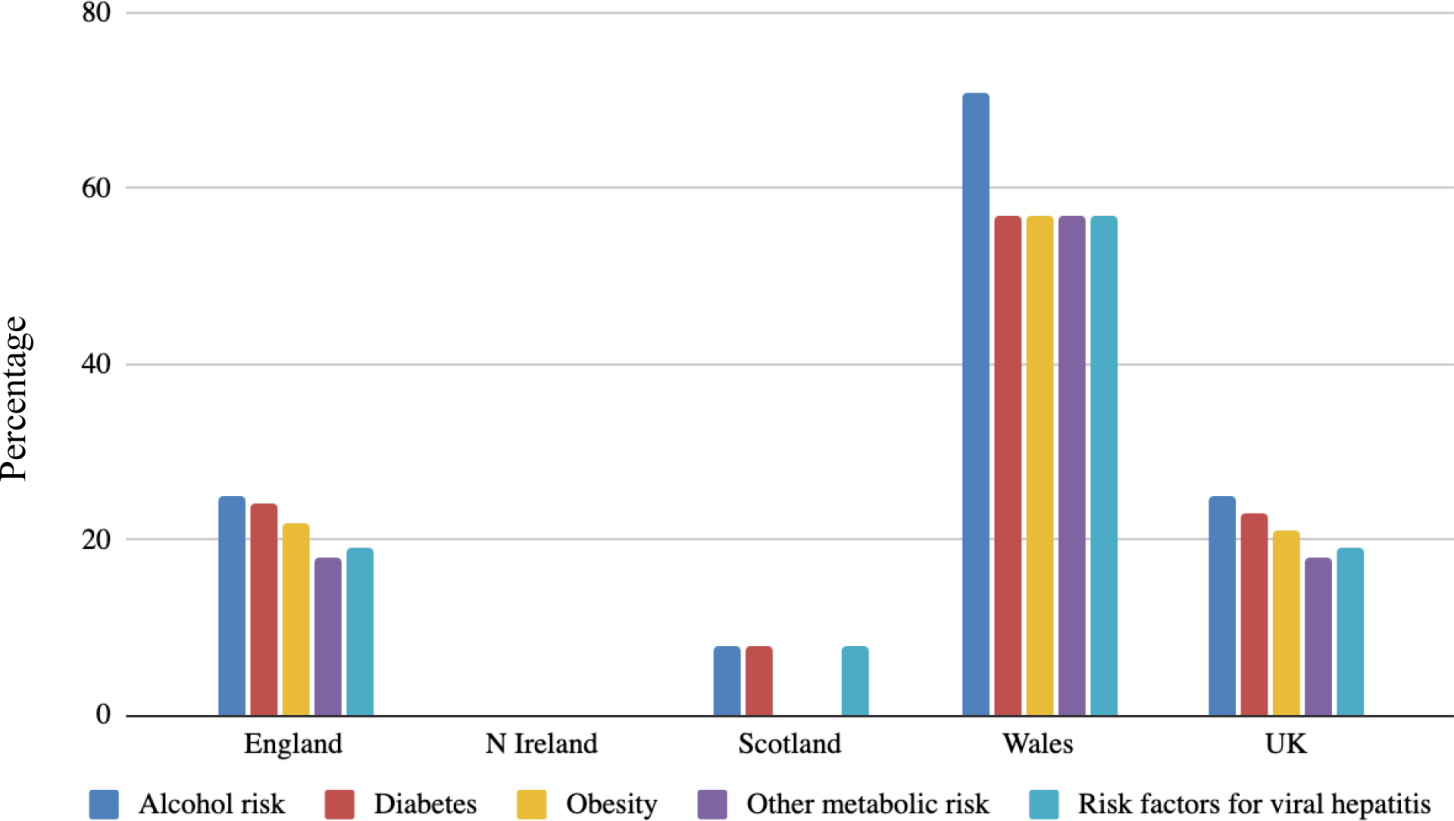
Percentage of clinical commissioning group or health authorities using proactive methods to identify liver disease by risk factor

**Figure 3.**
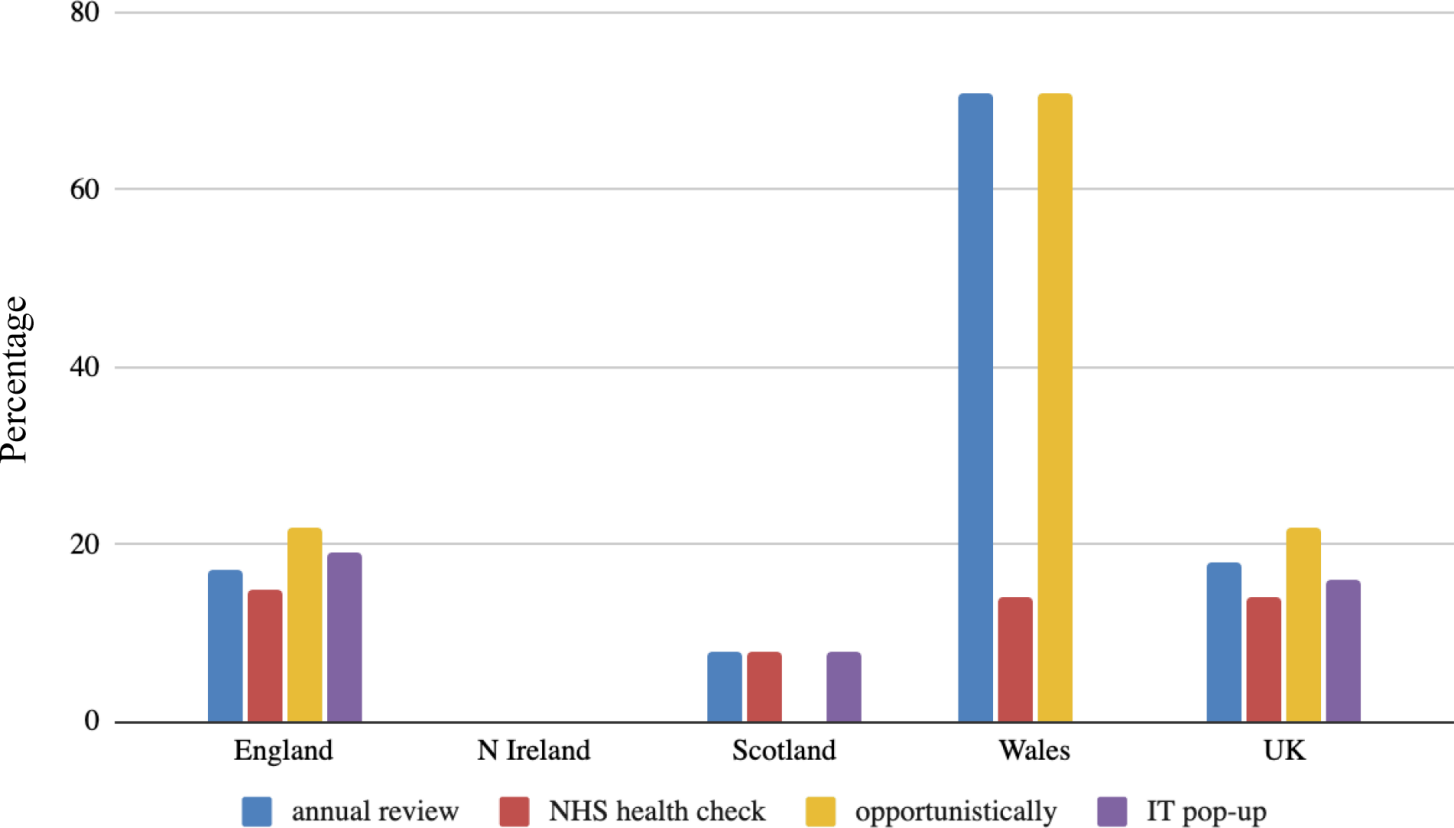
Percentage of clinical commissioning groups and health authorities using proactive methods to identify liver disease by assessment method

Proactive assessment of those with known liver disease risk factors to detect disease was endorsed by around one-quarter (38/159, 24%) of CCGs and health boards. The most common risk factors taken forward for assessment were alcohol risk and diabetes. There was marked variation between regions in the use of these risk factors to detect liver disease with the most comprehensive risk factor-based strategies endorsed by the Welsh health boards (Figure 2). Of those who did proactively look for liver disease using a risk factor-based approach, common methods for identifying these patients were at already scheduled annual reviews (for other conditions such as diabetes, hypertension), NHS health checks, as well as opportunistically, and using developed IT-system pop-up (Figure 3).

The survey allowed for free-text comments to clarify and explain responses. Of note in the results presented is the lack of health board engagement in any of the response areas by those responding from Northern Ireland. Free-text responses clarified that Northern Ireland is in the process of working on a nationwide response to liver disease (liver disease is under the remit of a national specialist services commissioning team), including endorsing a computer-assisted decision-making tool for assessment of abnormal liver blood tests (based on a model developed in Tayside, Scotland^13^), as well as a NAFLD pathway, including fibrosis assessment, using indirect serum markers and FibroScan. This work has been delayed owing to the impact of COVID-19.

## Discussion

### Summary

This survey represents the only national comprehensive overview of commissioning for community management of liver disease to date.

It reveals variable commissioner and health board engagement with liver disease across the UK, in all of the areas surveyed. Overall, there were low levels of engagement across many areas from having a named liver disease lead, to commissioning services to allow for evidence-based management of people at risk of and with liver disease. There was marked regional variation, with Wales being the only nation with high levels of engagement in all areas surveyed.

### Strengths and limitations

The limitations of a cross-sectional survey approach are well documented and this snapshot relied on the survey reaching a responder able to answer the survey questions accurately within the CCG or health board. The cross-checking with related providers, as well as the clear survey introduction in correspondence with all invitees, minimised this potential limitation. It is also acknowledged that individual GP practices may use their own templates and follow national guidelines to manage liver disease, independent of any CCG or health board recommendations. The survey results should thus be interpreted with caution when using them as a representation of individual clinician practice, although availability of diagnostics and the normalisation of pathways is likely to be driven at a regional level, meaning variation in individual practice is likely limited by these factors.

### Comparison with existing literature

Although there is no directly comparable literature on commissioning of liver services in the UK, there are other data supporting the finding of stark and unacceptable regional variation in care for patients with liver disease. Public Health England (PHE) published the second *Atlas of Variation* in risk factors and health care for liver disease in England in 2017 and showed marked variation by CCG in levels of hospital admissions for liver disease (8.5 fold differences by CCG) and under 75-year mortality rates (7.7 fold differences).^14^ The present study highlights a stark contrast in England between these clinical-burden measures and the managerial resource applied to liver disease. Although CCG boundaries have changed since the publication of the 2017 atlas, the lack of geographical matching between under 75-year mortality or hospital admissions for liver disease and the factors measured in the survey is clear, with high-mortality areas certainly being no more likely to have leadership or pathways in place (Supplementary Figure S1). Of the 15 English CCGs with the highest under 75-year mortality rates, 10 still have the same geographical boundaries. Only two have a named lead for liver disease, only four have liver pathways, and none have a means of regularly reviewing their liver disease data. There have also been surveys looking at liver services from the provider perspective, again revealing marked regional variation in the provision of standardised pathways of care and access to specialist services.^15,16^ Although this existing work differs from this survey in scope and outcomes studied, all this work supports the existence of a ‘postcode lottery’ in both health services available and outcomes for people with liver disease in the UK.

### Implications for practice

The findings highlight the tangible difference that a regional policy initiative can make. The Welsh National Liver Plan ran from 2015–2020, a Welsh government response to the rising morbidity and mortality from liver disease.^17^ One of the six delivery themes of the plan was that people with liver disease should be detected early and referred for treatment. Work within this theme included developing standardised referral pathways, with GP clinical champions working with health board liver disease teams to improve risk management, detection, and referral pathways. The stark difference between Wales and other nations of the UK within responses to this survey should act to incentivise policymakers to adopt and standardise evidence-based care for people with liver disease in their local populations.

CCGs in England are to be gradually phased out over the next year, with much of their remit being subsumed into new Integrated Care Systems (ICS) covering wider populations and geographies.^18^ The findings of this survey, far from being redundant as a consequence of this, can provide valuable lessons moving forward. The ICS model is in many ways more aligned to the health board model in other UK nations, with the opportunity to reduce inequalities in care and outcomes for liver disease if a truly regional, integrated, evidence-based approach to detection and management is taken. A less fragmented system should provide opportunity for those commissioners with already well-developed community liver services in place to lead within new ICS areas, spreading effective practice rather than the need to develop new models. Royal College of General Practitioners-endorsed national liver commissioning guidelines provide a recommended defined standard of care to work towards.^19^ Promoting these standards and considering incentivisation through QOF should be considered as further effective methods to drive change in the commissioning of community liver disease services, in response to these findings and other work.

This survey provides a UK-wide overview of system leadership of community liver services. The low levels of engagement and marked variation should be of interest to primary care practitioners, liver specialists, and policymakers. In an area of health care where there is a rising disease burden, the results of this survey should be used as a catalyst to drive change, reduce healthcare variation, and highlight necessary improvements to the primary care management of chronic liver disease in the UK.
